# Exogenous miRNAs induce post-transcriptional gene silencing in plants

**DOI:** 10.1038/s41477-021-01005-w

**Published:** 2021-10-14

**Authors:** Federico Betti, Maria Jose Ladera-Carmona, Daan A. Weits, Gianmarco Ferri, Sergio Iacopino, Giacomo Novi, Benedetta Svezia, Alicja B. Kunkowska, Antonietta Santaniello, Alberto Piaggesi, Elena Loreti, Pierdomenico Perata

**Affiliations:** 1 PlantLab, Institute of Life Sciences, Sant’Anna School of Advanced Studies, Pisa, Italy; 2 NEST, Scuola Normale Superiore, Pisa, Italy; 3 Department of Biology, University of Pisa, Pisa, Italy; 4 Valagro SpA, Atessa, Italy; 5 Institute of Agricultural Biology and Biotechnology, National Research Council, Pisa, Italy

**Keywords:** Plant signalling, Plant physiology

## Abstract

Plants seem to take up exogenous RNA that was artificially designed to target specific genes, followed by activation of the RNA interference (RNAi) machinery. It is, however, not known whether plants use RNAs themselves as signalling molecules in plant-to-plant communication, other than evidence that an exchange of small RNAs occurs between parasitic plants and their hosts. Exogenous RNAs from the environment, if taken up by some living organisms, can indeed induce RNAi. This phenomenon has been observed in nematodes and insects, and host *Arabidopsis* cells secrete exosome-like extracellular vesicles to deliver plant small RNAs into *Botrytis cinerea*. Here we show that micro-RNAs (miRNAs) produced by plants act as signalling molecules affecting gene expression in other, nearby plants. Exogenous miRNAs, such as *miR156* and *miR399*, trigger RNAi via a mechanism requiring both AGO1 and RDR6. This emphasizes that the production of secondary small interfering RNAs is required. This evidence highlights the existence of a mechanism in which miRNAs represent signalling molecules that enable communication between plants.

## Abstract (web summary)

This study shows that miRNAs produced by plants act as signalling molecules that affect gene expression in nearby plants. This RNAi induced by exogenous miRNAs enables communication between plants and requires the production of secondary siRNAs.

## Main

Small RNAs (sRNAs) are 21- to 24-nucleotide non-coding molecules that affect a variety of developmental and physiological processes in plants^1^. There are two groups of sRNAs, based on their biogenesis and mode of action. Micro-RNAs (miRNAs) belong to the first group, which comprises sRNAs encoded by specific genes producing a single-stranded, self-complementary, non-coding RNA that forms a hairpin structure subsequently processed by Dicer-like proteins (DCL) to produce a 21- to 22-nucleotide mature miRNA. The second group includes small interfering RNAs (siRNAs), which result from DCL-dependent cleavage of a double-stranded RNA molecule synthesized by RNA-DEPENDENT RNA POLYMERASE6 (RDR6). Single-stranded sRNAs are loaded onto ARGONAUTE (AGO) proteins belonging to the RNA-induced silencing complex. miRNAs are predominantly loaded onto the AGO1 protein. The RNA-induced silencing complex exploits the sRNA in a sequence-homology dependent manner to recognize mRNA targets, which are cleaved and eventually degraded^2^.

Within a plant, sRNAs act locally, but can also move from one cell to another, presumably via the plasmodesmata, and are transported systemically over long distances via the vasculature^3,4^. Gene silencing triggered by sRNAs can eventually spread throughout an entire plant. How these sRNAs move within the plant is still unclear. *miR399*, which is produced in response to phosphate starvation, is present in the phloem sap of several plants and has been demonstrated to be a phloem-mobile microRNA^5^. In phosphate-starved plants, expression of *miR399* is increased, leading to *miR399* translocation to the root system where it affects expression of its target, namely the E2 conjugase PHO2 which modulates degradation of the phosphate transporter PHO1 (ref. ^6^). Therefore, *miR399* translocation to the root system in phosphate-starved plants leads to activation of PHO1 and increased phosphate uptake.

Another well-studied miRNA proposed to be phloem-mobile is *miR156* (ref. ^7^). *miR156* regulates several developmental traits by repressing *SQUAMOSA-PROMOTER BINDING PROTEIN-LIKE* (*SPL*) transcription factors. Among the processes regulated by the *miR156/SPL* module it is worth citing juvenile-to-adult transition in *Arabidopsis*. Overexpressors of *miR156* flower extremely late as a consequence of an extended juvenile phase^8^.

Interestingly, sRNAs are not only mobile signalling and regulatory molecules within the plant, but also move between plants and interacting organisms, including pathogens, to induce gene silencing. This phenomenon is known as cross-kingdom/organism RNA interference (RNAi)^9^. In response to infection with *Verticillium dahliae*, cotton plants display increased production of *miR166* and *miR159*, and export both to the fungal hyphae to silence two *Verticillium* genes that are essential for fungal virulence^10^. *Botrytis cinerea* delivers its sRNAs into plant cells to silence host immunity genes^11^, and host *Arabidopsis* cells secrete exosome-like extracellular vesicles to deliver plant sRNAs into *B. cinerea*^12^. Exogenous RNAs may either be taken up by the fungal cells with which they come into contact with on the leaf surface or be taken up by plant cells first and then transported into fungal cells^13^. Interestingly, locally sprayed double-stranded RNAs (dsRNAs) also inhibit pathogen virulence at distal, non-treated leaves^13,14^. This suggests that these artificially synthesized dsRNAs spread systemically within plants after external application on the leaf surface.

sRNAs are also exchanged between plants. Dodders (*Cuscuta* spp.), an obligate parasitic plant, uses haustoria to obtain water and nutrients from its host plant. *Cuscuta campestris* haustoria accumulate high levels of many 22-nucleotide miRNAs targeting *Arabidopsis thaliana* messenger RNAs during parasitism, resulting in mRNA cleavage, secondary siRNA production and decreased mRNA accumulation^15^. These results show that miRNAs from dodders act as trans-species modulators of host-gene expression, suggesting that they influence the virulence of parasitic plants.

If exogenous RNAs from the environment are taken up by some living organisms, they can induce RNAi. This phenomenon, called ‘environmental RNAi’, has been observed in nematodes and insects^16^, but not in plants. However, topical application of laboratory-synthesized dsRNAs targeting insect developmental genes impairs insect growth^17,18^. These results indicate that gene expression within insect pests is suppressed through the uptake of either dsRNAs or sRNAs; however, it is unclear whether the RNA taken up by insects was inside the treated plant or present on its surface.

Some miRNAs can act systemically in the plant, indicating that they behave as mobile signalling molecules^4^. However, it is not known whether they are present in the environment, whether they are taken up by plants or whether this ultimately leads to post-transcriptional silencing of miRNA target genes in the receiving plant.

Here, we demonstrate that exogenous miRNAs can induce post-transcriptional gene silencing (PTGS), and that transfer of miRNA between neighbouring plants can influence gene expression. We utilized two miRNA modules, namely miR156/SPL and miR399/PHO2, because both have been reported to be cell mobile, regulate well-characterized plant processes and induce the synthesis of secondary siRNAs, potentially amplifying initial miRNA-dependent cleavage of the target mRNA^19^.

## Results

### Exogenous miRNAs trigger silencing of target genes

**Fig. 1:**
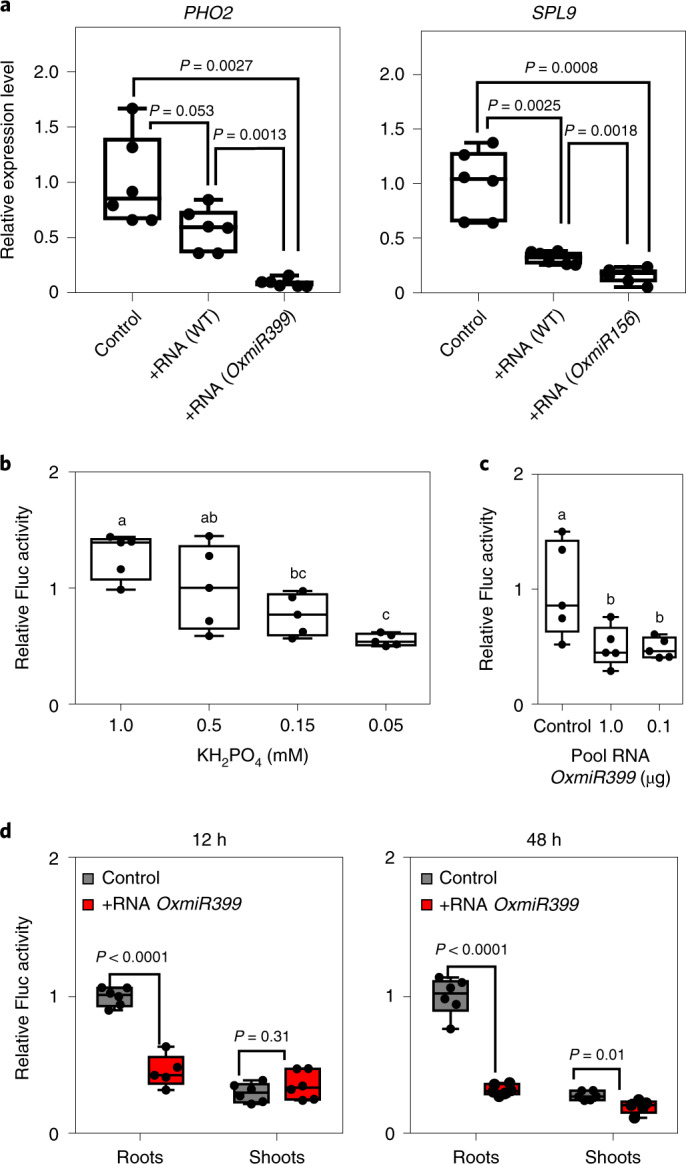
Exogenous miRNAs influence the expression of their target genes in the receiving seedlings. **a**, Effect of exogenous total RNA (1 μg, added to a total volume of 2 ml), extracted from wild-type (WT) plants or from plants overexpressing either *miR399* or *miR156*, on the expression of *PHO2* and *SLP9* in *Arabidopsis* seedlings (8 days old). **b**, Response of *pPHO2:Fluc* seedlings (6 days old) to phosphate starvation. **c**, Response of *pPHO2:Fluc* seedlings (6 days old) to exogenous RNA from *OxmiR399* plants (added to a total volume of 2 ml). **d**, Response to exogenous RNA (0.1 μg) from *OxmiR399* plants (added to a total volume of 2 ml) in roots and shoots of *pPHO2:Fluc* seedlings (6 days old) grown in 2 ml of liquid medium. For all panels, the bottom and top of each box denote the first and third quartile, respectively. **a**, *n* = 6 biological replicates; **b**–**d**, *n* = 5 biological replicates. In the boxplots, dots represent single data points, whiskers denote the minimum/maximum values, the box defines the interquartile range, the centre represents the median, and box bounds represent the lower and upper quartiles. Welch’s *t*-test (two-sided) values are shown. Different letters (a,b,c) indicate differences in ANOVA tests (Tukey’s post‐hoc test, *P* < 0.05). Source data

**Extended Data Fig. 1:**
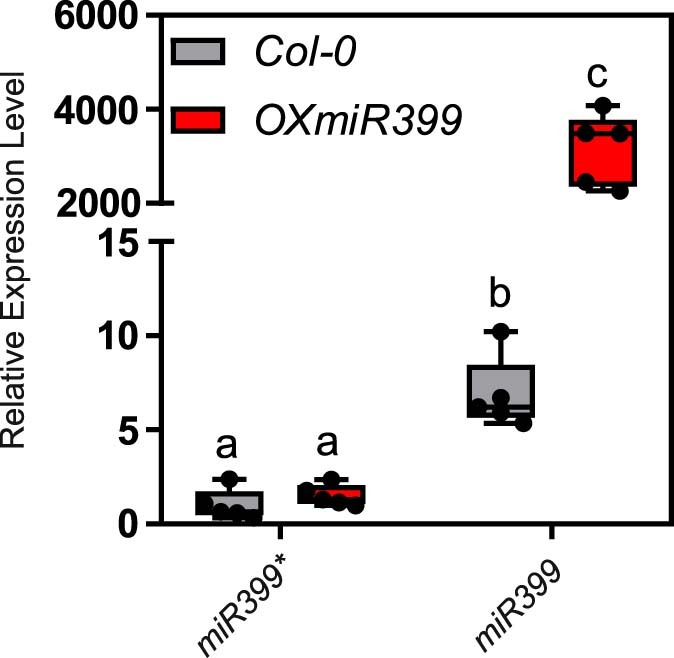
Ratio *miR399*/miR399* in RNA extracts from wild-type plants (*Col-0*) and plants over-expressing *miR399*. The level of *miR399* and miR399* (passenger strand) was determined by stem-loop qPCR using specific primers. In the box-blots, the dots represent the single data points, whiskers denote the min/max values, the box defines the interquartile range, centre represents the median, box bounds represent the lower and upper quartiles (n = 5 biological replicates). Different letters (a,b,c) refer to differences in ANOVA tests (Tukey’s post‐hoc test, P < 0.05). Source data

We explored the possibility that miRNAs can silence their target genes when applied exogenously to *Arabidopsis* seedlings grown in vitro. RNA was extracted from wild-type and miRNA-overexpressing plants (*miR399* and *miR156*) and added to the liquid medium in which wild-type *Arabidopsis* seedlings were grown. The extracted RNA contains predominantly the guide strand of the miRNA, whereas the passenger strand, although present, is less abundant (Extended Data Fig. 1). After 24 h of incubation, expression of the respective targets of *miR399* and *miR156*, *PHO2* and *SPL9*, was analysed (Fig. 1a). The data showed repression of *PHO2* and *SPL9* in seedlings treated with exogenous RNA. Interestingly, RNA extracts enriched in either *miR399* or *miR156* had a stronger repressive effect on their mRNA targets compared with RNA extracts from the wild-type (Fig. 1a). These results suggest that exogenous miRNAs trigger RNAi in the receiving plant. To ensure that the observed effect could be assigned unequivocally to an RNAi mechanism, we produced transgenic plants expressing the reporter gene Firefly luciferase (*Fluc*) bearing the *miR399* target sequence from *PHO2* under control of the *PHO2* promoter (named *pPHO2:Fluc*). In these plants, the level of luciferase (LUC) is expected to respond to phosphate starvation, given that the stability and translation of *LUC* is determined by the *miR399* target sequence. This was demonstrated experimentally by treating seedlings with decreasing P-availability which, as expected, reduced LUC activity accordingly (Fig. 1b). We then treated the *pPHO2:Fluc* seedlings with an RNA extract enriched in *miR399* and found that LUC activity was repressed when exogenous RNA was fed to the seedlings (Fig. 1c). These results indicated that the repression of *PHO2* mRNA observed in Fig. 1a can be assigned to *miR399*, which interacts with the *miR399* target sequence. We explored the repressive effects of *miR399*-enriched RNA extracts on roots and shoots of *Arabidopsis* seedlings. The results highlighted a stronger and faster response in roots, although the effect was also visible in shoots 48 h after the start of treatment (Fig. 1d).

### Exogenous miRNAs affect the phenotype of the receiving plant

**Fig. 2:**
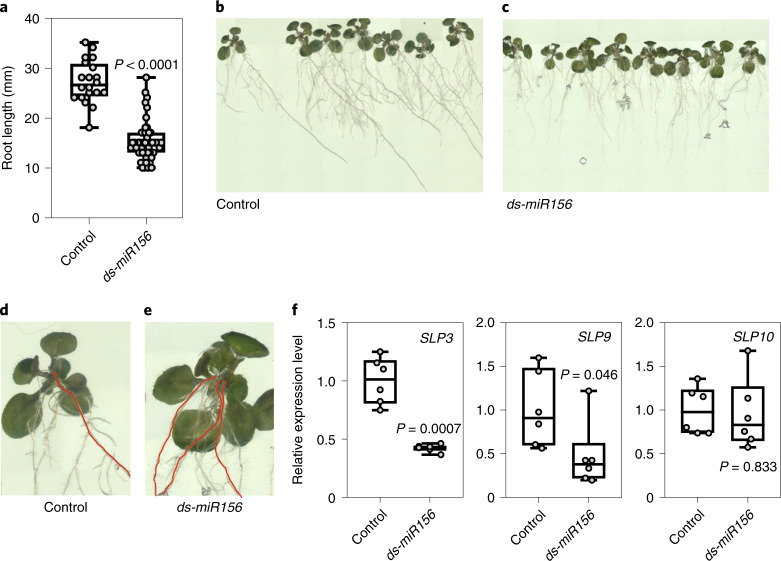
Exogenous *ds-miR156* influences the root phenotype in *Arabidopsis* seedlings. **a**, Primary root length in 10-day-old seedlings germinated on control medium (*n* = 20) or on a medium supplemented with 0.2 μM synthetic *ds-miR156* (*n* = 48). **b**,**c**, Photographs of representative control (**b**) and *ds-miR156*-treated (**c**) vertical plates (15 days after sowing). **d**,**e**, Magnified seedlings from **b** (**d**) and **c** (**e**) showing (in red) the presence of adventitious roots. **f**, Transcript level of *SPL3*, *SPL9* and *SPL10* extracted from roots at the stage depicted in **b** and **c**. For all boxplots, the bottom and top of each box denote the first and third quartile, respectively (*n* = 6 biological replicates). In the boxplots, dots represent single data points, whiskers denote minimum/maximum values, the box defines the interquartile range, the centre represents the median and box bounds represent the lower and upper quartiles. Welch’s *t*-test (two-sided) values are shown. Source data

*miR156* has an array of effects in plants, one of which is a clear influence on root architecture^20,21^. We tested whether germinating *Arabidopsis* seeds on a medium containing a chemically synthesized, pure *miR156* (referred to as synthetic *miR156*) would result in a phenotype compatible with the role of this miRNA in root development. Development of the primary root was clearly inhibited by synthetic *miR156* after 10 days of germination (Fig. 2a), in line with the reduced primary root growth observed in a transgenic line overexpressing *miR156a*^21^. Later, inhibition of the primary root elongation was retained, together with the development of adventitious roots (Fig. 2b,c,d,e). Remarkably, the *miR156/SPL* module has been shown to be responsible for the development of crown roots in rice^22^. The regulation of root architecture by the *miR156/SPL* module in *Arabidopsis* relies on the repression of three *SPL* genes involved in root development, namely *SPL3*, *SPL9* and *SPL10* (ref. ^20^). We analysed the expression levels of these three *SPL* genes in roots collected at the end of the experiment shown in Fig. 2b,c. *SPL3* was strongly repressed by the presence of exogenous, synthetic *ds*-*miR156*, whereas repression of *SPL9* was modest. *SPL10* expression was unaffected by the treatment (Fig. 2f). Overall, the results indicate that exogenous *ds*-*miR156* is able to modulate root architecture by adjusting the expression of *SPL3*.

### Exogenous miRNAs are translocated by the xylematic route

**Fig. 3:**
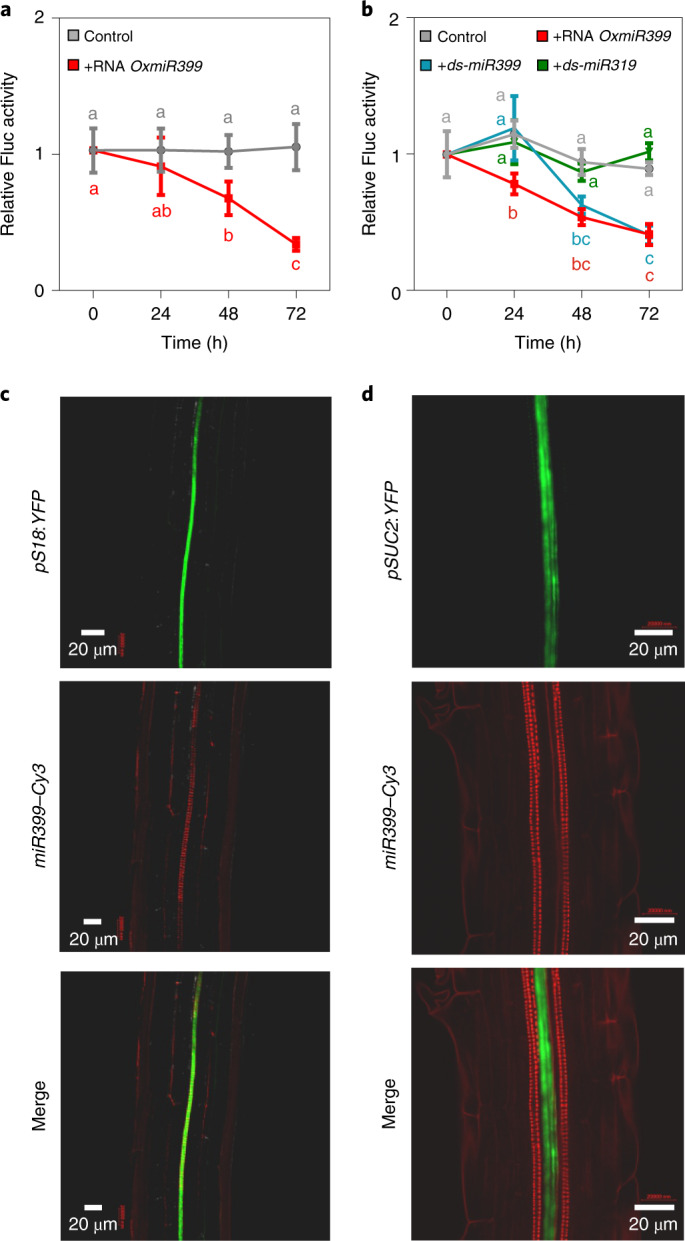
Translocation and response to exogenous miRNAs. **a**, Time-course of the response of *pPHO2:Fluc* seedlings (6 days old) to exogenous RNA (0.1 μg) from *OxmiR399* plants (added to a total volume of 2 ml). Data are mean ± s.d. (*n* = 5 biological replicates). **b**, Time-course of the response of *pPHO2:Fluc* seedlings (6 days old) to exogenous RNA (0.1 μg) from *35S:miR399* plants (added to a total volume of 2 ml) and to chemically synthesized *ds-miR399* and *ds-miR319* (0.2 nM). Data are mean ± s.d. (*n* = 5 biological replicates). **c**, Translocation of Cy3-labelled *miR399* (*miR399–Cy3*) in *Arabidopsis* *pS18:YFP* (showing YFP-labelled xylem) seedlings (6 days old). Seedlings were incubated with *miR399–Cy3* for 2 h and subsequently observed using a confocal microscope. **d**, Translocation of Cy3-labelled *miR399* (*miR399–Cy3*) in *Arabidopsis* *pSUC2:YFP* (with YFP expressed in the phloem) seedlings (6 days old). Seedlings were incubated with *miR399–Cy3* for 2 h and subsequently observed using a confocal microscope. Welch’s *t*-test (two-sided) values are shown in **a** and **b**. Different letters (a,b,c) indicate differences in ANOVA tests (Tukey’s post‐hoc test, *P* < 0.05). The experiments reported in **c** and **d** were repeated three times with consistent results. Source data

We studied the efficacy of a chemically synthesized, pure *miR399* (referred to as synthetic *miR399*) compared with a *miR399*-enriched RNA plant extract. The seedlings responded to the *miR399*-enriched RNA plant extract with repression being evident after 48–72 h (Fig. 3a). We used this time frame to compare the plant-derived extract (enriched in *miR399*) with two synthetic miRNAs, namely *ds-miR399* and *ds-miR319*; the latter was used as a negative control. First, we checked that synthetic *ds-miR399* triggers *Fluc*-repression in *pPHO2:Fluc* seedlings. In this experiment, we observed repression of *Fluc* by both the plant-derived RNA extract and synthetic *ds-miR399*, which was evident after 48 h. Synthetic *ds-miR319* did not trigger repression of *Fluc* activity (Fig. 3b). These results indicated that a synthetic version of *ds-miR399*, with a sequence identical to the mature *miR399* present in plants, is sufficient to trigger repression of *Fluc* acting on the *PHO2*-derived *miR399* target sequence.

Second, we checked the actual uptake of miRNAs in treated seedlings using synthetic *ds-miR399* labelled with Cy3 as a fluorescent probe. To distinguish between the phloematic and xylematic routes of possible in planta translocation of exogenous miRNAs, we used *pSUC2:YFP* and *pS18:YFP* plants in which yellow fluorescent protein (YFP) labels the phloem and xylem, respectively^23^. Analysis of confocal microscopy images of seedlings treated with exogenous *miR399*–Cy3 revealed a clear overlap of Cy3 fluorescence with YFP in *pS18:YFP* plants (Fig. 3c) but not *pSUC2:YFP* (Fig. 3d), indicating that exogenous *miR399* is preferentially translocated via the xylematic route.

### Exogenous *miR399* targets the *PHO2* RNA sequence

**Extended Data Fig. 2:**
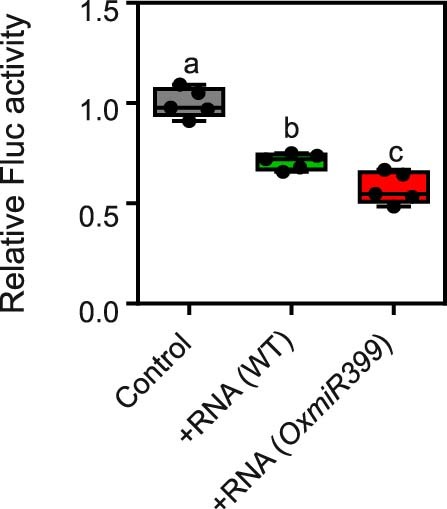
Dose response in protoplasts transformed with the *pUBQ10:PHO2-UTR-Fluc*. Protoplasts transformed with the *pUBQ10:PHO2-UTR-Fluc* were incubated with an RNA extract (0.1 μg) from wild-type plants or enriched in miR399. In the box-blots, the dots represent the single data points, whiskers denote the min/max values, the box defines the interquartile range, centre represents the median, box bounds represent the lower and upper quartiles (n = 5 biological replicates). Different letters (a,b,c) refer to differences in ANOVA tests (Tukey’s post‐hoc test, P < 0.05). Source data

We used protoplasts to investigate the mode of action of exogenous miRNAs. We isolated protoplasts from wild-type *Arabidopsis* plants and transiently transformed them with the construct *pUBQ10:PHO2-UTR–Fluc*, expressing a reporter mRNA containing the *Fluc* open reading frame fused to the 5′-untranslated region (UTR) of *PHO2* bringing the miR399-target sites, plus a *pUBQ10:Rluc* vector as an internal control. Protoplasts were then incubated with an RNA extract from wild-type plants or plants enriched in *miR399*. The results, shown in Extended Data Fig. 2, highlight that *miR399*-enriched RNA reduced *Fluc* activity in *pUBQ10:PHO2-UTR–Fluc* with an effect that is stronger than that of RNA extract from a wild-type donor plant. It is not surprising that RNA from the wild-type plant could repress *Fluc* activity given that *miR399* is quite well expressed in wild-type plants.

**Fig. 4:**
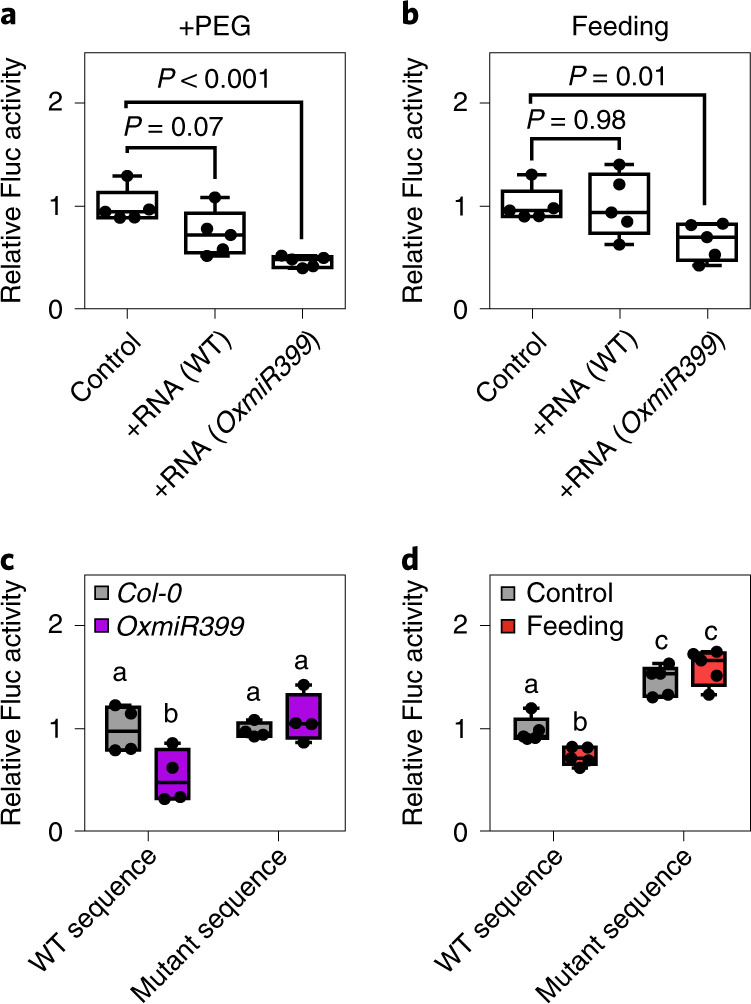
Responses to exogenous RNA in protoplasts from *pUBQ10:PHO2-UTR–Fluc* leaves. **a**, Effect of exogenous RNA (0.1 μg) from WT and *OxmiR399* plants (added to a total volume of 2 ml) in *pUBQ10:PHO2-UTR–Fluc* protoplasts treated with PEG 4000 to facilitate entry of the miRNAs (*n* = 5 biological replicates). **b**, Feeding of exogenous RNA (0.1 μg) from WT and *OxmiR399* plants to *pUBQ10:PHO2-UTR–Fluc* protoplasts (added to a total volume of 2 ml) (*n* = 5 biological replicates). **c**, Transformation of protoplasts from *OxmiR399* and *Col-0* plants with *pUBQ10:PHO2-UTR–Fluc* or *pUBQ10:random-UTR–Fluc* (*n* = 4 biological replicates). **d**, Feeding of exogenous RNA (0.1 μg, added to a total volume of 2 ml) from *OxmiR399* plants to WT protoplasts transformed with *pUBQ10:PHO2-UTR–Fluc* or *pUBQ10:random-UTR–Fluc* (*n* = 5 biological replicates). In the boxplots, dots represent single data points, whiskers denote minimum/maxmum values, the box defines the interquartile range, the centre represents the median and box bounds represent the lower and upper quartiles. Welch’s *t*-test (two-sided) values are shown. Different letters (a,b,c) indicate differences in ANOVA tests (Tukey’s post‐hoc test, *P* < 0.05). Source data

**Extended Data Fig. 3:**
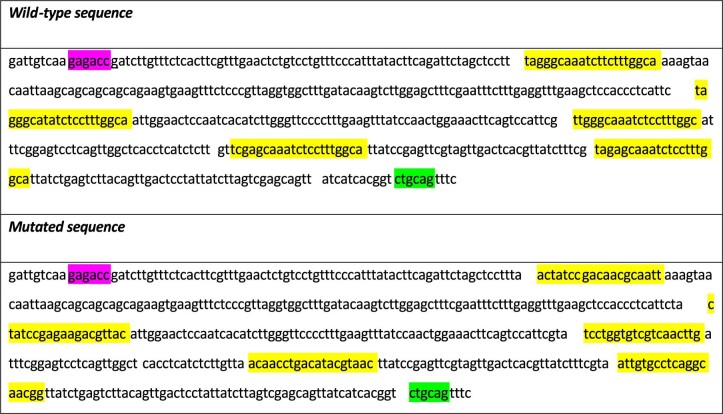
Wild-type and mutated sequences of 5’UTR of *PHO2* (726 to 219). Restriction sites of *PstI* and *BsaI* are highlighted in purple and green, respectively. Wild-type miR399-target sequences and the corresponding mutated versions are highlighted in yellow. The reported sequence is located between nucleotide 726 and nucleotide 219 upstream of the *PHO2* Met start codon.

Although polyethylene glycol (PEG) treatment to permeate the protoplast enhanced the efficacy of the RNA treatment (Fig. 4a), repression of *Flu*c was still observed with a normal feeding procedure (Fig. 4b). The protoplast system enabled us to transiently transform protoplasts with different versions of the *PHO2* target RNA sequence. We produced a version of the LUC reporter with the target RNA sequence showing a scrambled sequence that replaced the sequence recognized by *miR399* (Extended Data Fig. 3) and compared the wild-type target sequence with the mutated one. When transformed into protoplasts obtained from plants of the *35S:miR399* overexpressor, the *pUBQ10:PHO2-UTR–Fluc* construct bearing the wild-type *miR399* target RNA sequence drove repression of LUC, whereas the mutated version did not (Fig. 4c). Similarly, when wild-type protoplasts were transformed with the two LUC reporter sequences and fed exogenous *miR399*-enriched RNA, repression of LUC was observed only when the LUC sequence was associated with the wild-type *PHO2* target sequence (Fig. 4d).

**Extended Data Fig. 4:**
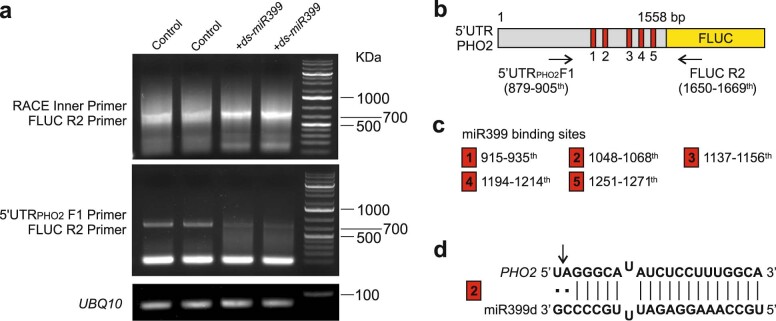
Detection of *miR399*-guided cleavage products of PHO2 by 5’RACE-PCR in pPHO2:Fluc seedlings incubated with *ds-miR399*. **a**. Semi-quantitative 5′RACE-PCR to detect 3′ cleavage fragments generated through miR399-guided cleavage of *PHO2* transcript. Starting from the left: no treated samples (control), samples incubated with *ds-miR399* (0.2 nM), the DNA ladder with standard sizes labeled (KDa). *UBQ10* was used as a loading control. **b**. Schematic representation of *5’UTR PHO2* sequence, the location of *miR399*-binding sites and positions of primers used in panel a. Arrows indicate primers orientation. **c**. Targeting sites of *miR399*. Numbers in red rectangles correspond to locations indicated in panel b. **d**. Alignment of *miRNA399* and its second targeting site on *PHO2*. Dots and vertical lines correspond to uncomplimentary and complementary nucleotides, respectively. The arrow shows the cleavage site of *miRNA399* in the *PHO2* sequence, which was detected after sequencing of 5’RACE-PCR products. The experiment was repeated two times with consistent results. Source data

We performed semiquantitative 5′-rapid amplification of complementary DNA ends (5′-RACE) PCR with reverse transcription (RT–PCR) to detect 3′-cleavage products from *PHO2* in seedlings treated with exogenous RNA. If no *miR399*-mediated cleavage occurs, PCR products using paired primers flanking the *miR399*-binding sites should be obtained. Otherwise, specific 3′-cleavage products should be obtained using the Generacer-specific primer and the primer from the *Fluc* sequences downstream of the *miR399*-binding sites. The results showed a higher intensity band corresponding to the cleaved *PHO2* product in seedlings treated with exogenous RNA, whereas the intact *PHO2* mRNA sequence was clearly more abundant in the untreated, control plants (Extended Data Fig. 4a). Furthermore, sequencing of the 3′-cleavage products from *PHO2* revealed that cleavage of the *PHO2* transcript by treatment with exogenous RNA occurred at precisely the second *miR399*-binding site (Extended Data Fig. 4b–d). This is in line with the findings of Allen et al.^24^, who identified *miR399*-binding sites 2 and 3 as the predominant sites of *miR399*-guided cleavage. These results indicate that the mechanism by which exogenous RNA triggers repression of specific mRNA targets of miRNAs occurs via canonical cleavage of the target sequence.

### Exogenous miRNA-triggered RNAi requires AGO1 and RDR6

**Fig. 5:**
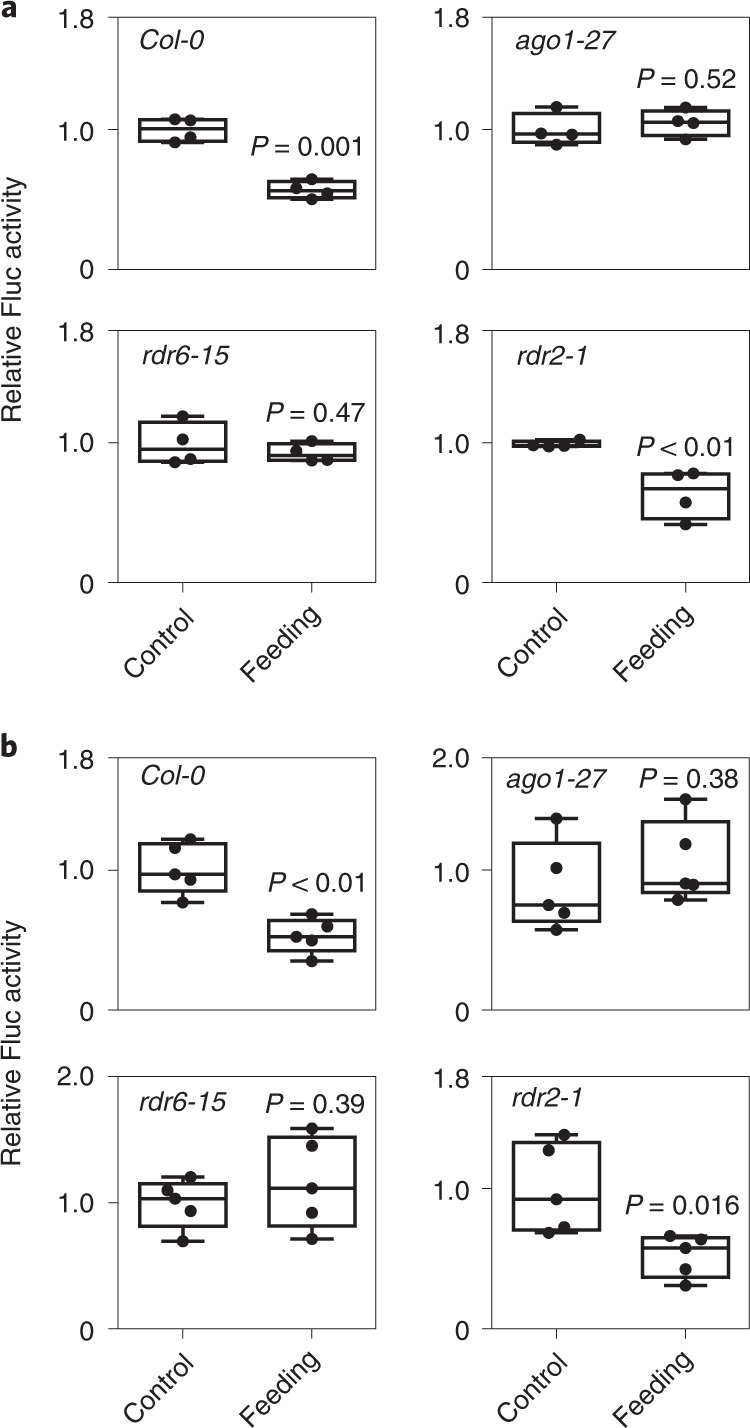
The exogenous *miR399* signalling pathway requires both AGO1 and RDR6. **a**, Feeding of exogenous RNA (0.1 μg, added to a total volume of 2 ml) from *OxmiR399* plants to protoplasts from WT (*Col-0*), *ago1-27*, *rdr6-15* and *rdr2-1* plants transformed with *pUBQ10:PHO2-UTR–Fluc* (*n* = 4 biological replicates). **b**, Feeding of exogenous synthetic *miR399* (0.2 nM) to protoplasts from WT (*Col-0*), *ago1-27*, *rdr6-15* and *rdr2-1* plants transformed with *pUBQ10:PHO2-UTR–Fluc* (*n* = 5 biological replicates). In the boxplots, dots represent single data points, whiskers denote minimum/maximum values, the box defines the interquartile range, the centre represents the median and box bounds represent the lower and upper quartiles. Welch’s *t*-test (two-sided) values are shown. Source data

**Extended Data Fig. 5:**
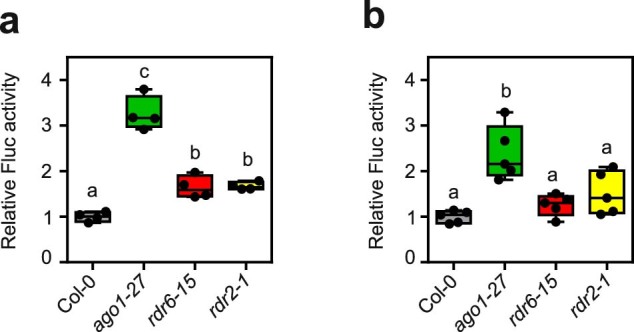
Fluc activity in protoplasts from wild-type (Col-0), *ago1-27*, *rdr6-15*, and *rdr2-1* plants and transformed with the *pUBQ10:PHO2-UTR-Fluc*. The activity of Fluc was higher in the *ago1-27*, as expected from the lack of repression of the PHO2 target sequence by the endogenous *miR399*. Panel **a** refers to the experiment reported in Fig. 5a, while panel **b** refers to the experiment reported in Fig. 5b. Both experiments were conducted without the addition of exogenous RNA. In the box-blots, the dots represent the single data points, whiskers denote the min/max values, the box defines the interquartile range, centre represents the median, box bounds represent the lower and upper quartiles. Different letters (a,b,c) refer to differences in ANOVA tests (Tukey’s post‐hoc test, P < 0.05). *t* (a: n = 4 biological replicates; b: n = 5 biological replicates). Source data

**Extended Data Fig. 6:**
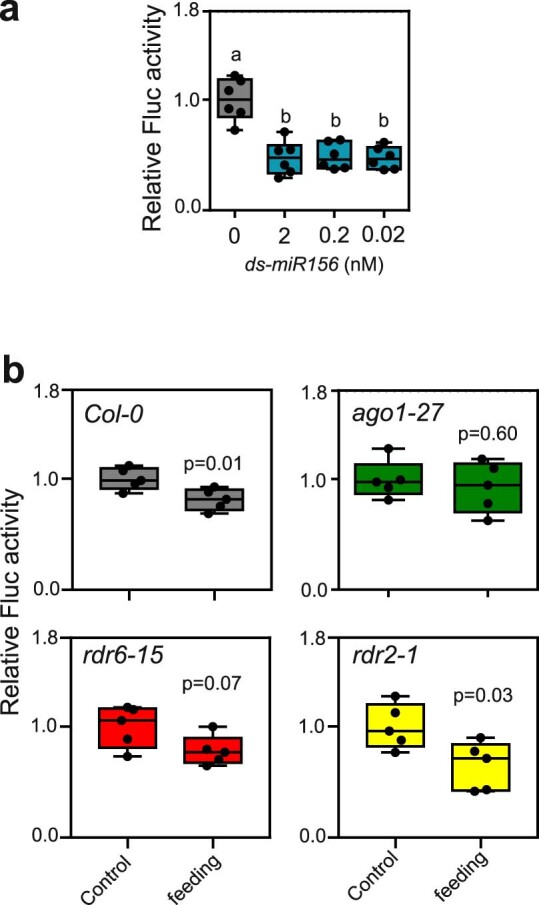
The exogenous *miR156* signalling pathway requires both AGO1 and RDR6. **a**. Feeding of exogenous synthetic *miR156* to wild-type protoplasts transiently transformed with *35S:SPL9-Fluc*, together with a *35S:Rluc* plasmid for normalization. **b**. Feeding of exogenous synthetic *miR156* (0.2 nM) to protoplasts from wild-type (Col-0), *ago1-27*, *rdr6-15*, and *rdr2-1* plants transformed with the *35S:SPL9-Fluc*, plus a *35S:Rluc* vector as internal control. In the box-blots, the dots represent the single data points, whiskers denote the min/max values, the box defines the interquartile range, centre represents the median, box bounds represent the lower and upper quartiles. Different letters (a,b) refer to differences in ANOVA tests (Tukey’s post‐hoc test, P < 0.05). Welch’s *t* test (two sided) values are shown (a: n = 6 biological replicates; b: n = 5 biological replicates). Source data

In plants, RNAi mediated by miRNAs requires the AGO1 protein. We prepared protoplasts from wild-type (*Col-0*), *ago1-27*, *rdr6-15* and *rdr2-1* plants and transformed them with the *pUBQ10:PHO2-UTR–Fluc*. The activity of *Fluc* was higher in *ago1-27* plants, as expected from the lack of repression of the PHO2 target sequence by the endogenous *miR399* (Extended Data Fig. 5). When wild-type protoplasts were compared with protoplasts obtained from leaves of the *ago1-27* mutant, repression of LUC after feeding with exogenous RNA was observed only in the wild-type, indicating that a functional AGO1 protein is required to trigger RNAi, thereby affecting the LUC transcript (Fig. 5a). *miR399* is one of the miRNAs that induces transitivity via a mechanism requiring RDR6 (refs. ^19,25^). When protoplasts were prepared from *rdr6-15* leaves, repression by exogenous RNA was abolished (Fig. 5a). When a RDR2 mutant was used (*rdr2-1*), repression of LUC occurred as in the wild-type (Fig. 5a). This was expected, given that RDR2 is an RNA-dependent direct polymerase involved in the biogenesis of endogenous 24-nucleotide siRNAs, and is thus involved in transcriptional gene silencing, but not in PTGS. The requirement for functional AGO1 and RDR6 proteins to trigger the silencing of genes bearing a miRNA target sequence was confirmed when the experiment was repeated using synthetic *ds-miR399* (Fig. 5b) and synthetic *ds-miR156* (Extended Data Fig. 6).

**Fig. 6:**
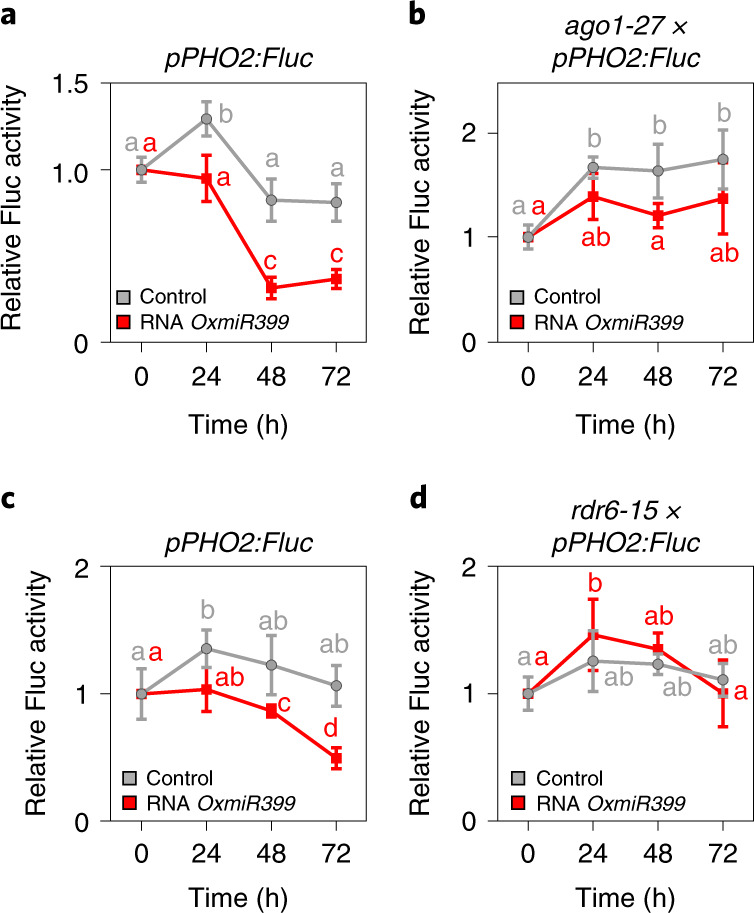
Exogenous RNA efficacy in seedlings of *pPHO2:Fluc*, *rdr6-15*×*pPHO2:Fluc* and *ago1-27*×*pPHO2:Fluc*. **a**, Seedlings of *pPHO2:Fluc* were fed with exogenous RNA extracted from *OxmiR399* plants (0.1 μg, added to a total volume of 2 ml). Data are mean ± s.d. (*n* = 6 biological replicates). **b**, Seedlings of *ago1-27*×*pPHO2:Fluc* were fed with exogenous RNA extracted from *OxmiR399* plants (0.1 μg, added to a total volume of 2 ml). Data are mean ± s.d. (*n* = 6 biological replicates). **c**, As in **a**, independent experiment as a control for **d**. Data are mean ± s.d. (*n* = 5 biological replicates). **d**, Seedlings of *rdr6-15*×*pPHO2:Fluc* were fed with exogenous RNA extracted from *OxmiR399* plants (0.1 μg, added to a total volume of 2 ml). Data are mean ± s.d. (*n* = 5 biological replicates). Different letters (a,b,c,d) indicate differences in ANOVA tests (Tukey’s post‐hoc test, *P* < 0.05). Source data

We then investigated whether the requirement for AGO1 and RDR6 to trigger RNAi after feeding exogenous RNA could be confirmed in planta. We crossed the *pPHO2:Fluc* reporter line with *ago1-27* and *rdr6-15* and used them to check whether exogenous RNA would repress expression of LUC in the resulting crosses. When seedlings of the *pPHO2:Fluc* reporter line were fed with a pool of RNA extracted from *35S:miR399* plants, repression of LUC activity was observed within 24–48 h after treatment (Fig. 6a,c). By contrast, *pPHO2:Fluc* crossed with *ago1-27* was insensitive to exogenous RNA (Fig. 6b), which was even more evident with *pPHO2:Fluc* crossed with *rdr6-15* (Fig. 6d).

**Extended Data Fig. 7:**
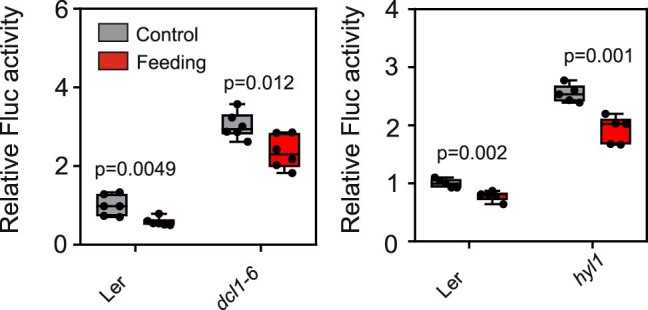
The exogenous *miR399* signalling pathway does not require DCL1 and HYL1. Feeding of exogenous synthetic *miR399* to wild-type protoplasts transiently transformed with the *pUBQ10:PHO2-UTR-Fluc* together with a *35S:Rluc* plasmid for normalization. The activity of Fluc was higher in the *dcl1-9* and in *hyl1*, as expected from the lack of repression of the *PHO2* target sequence by the endogenous *miR399*, which cannot be correctly processed in the absence of *DCL1* and *HYL1*. Feeding the synthetic *miR399* reduces the Fluc activity indicating successful repression of the PHO2 miR399-target sequence. In the box-blots, the dots represent the single data points, whiskers denote the min/max values, the box defines the interquartile range, centre represents the median, box bounds represent the lower and upper quartiles (n = 6 biological replicates). Welch’s t test (two sided) values are shown. Source data

We explored the dicer activity requirement for the observed effects of exogenous RNA. DICER1 (DCL1) and HYPONASTIC LEAVES 1 (HYL1) are required to ensure accurate cleavage of mature miRNA from the pre-miRNA stem–loop^26^. Protoplasts from wild-type (*Ler*) and *dcl1-9* plants transformed with *pUBQ10:PHO2-UTR–Fluc* display different levels of *Fluc* expression, with the *PHO2* target sequence being less repressed when the endogenous *miR399* is not produced properly due to defective *DCL1* and *HYL1* activity (Extended Data Fig. 7). Feeding protoplasts with synthetic *ds*-*miR399* reduced *Fluc* activity, indicating repression of the *PHO2* target sequence (Extended Data Fig. 7). This demonstrates that exogenous *miR399* can complement the defective endogenous processing of *miR399* caused by the absence of DCL1/HYL1.

### Secreted miRNAs influence gene expression in nearby plants

**Fig. 7:**
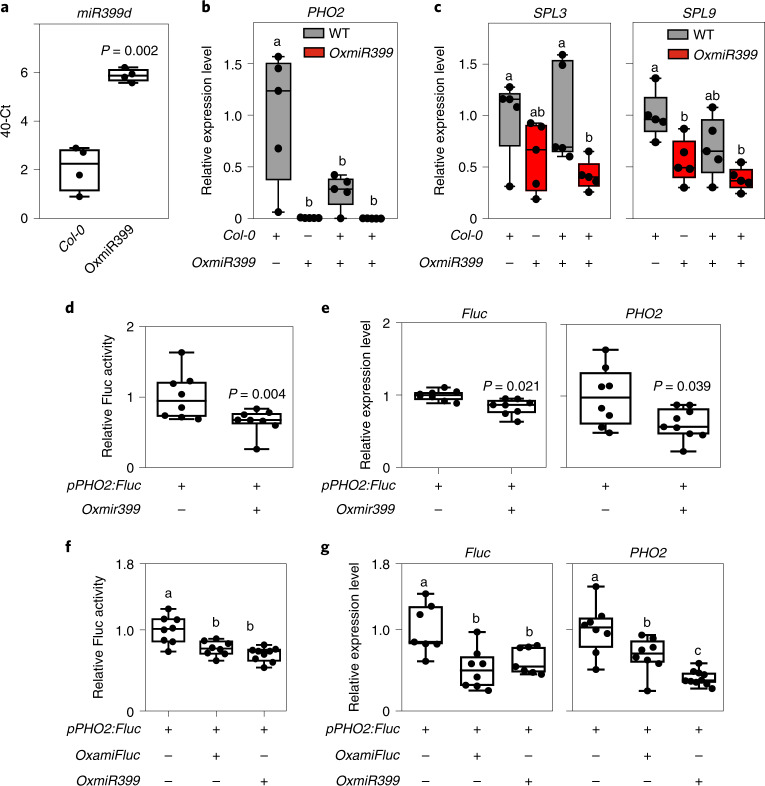
Plants overexpressing *miR399* influence the expression of *PHO2* in WT plants sharing the same hydroponic medium. **a**, Presence and quantity of *miR399* in the external medium of WT and *OxmiR399* plants (*n* = 4 biological replicates). The miRNA amount is expressed using the value resulting after subtracting the actual cycle threshold (Ct) value in PCR from the arbitrary value of 40 (40-Ct). **b**, Expression of *PHO2* in WT plants, *OxmiR399* plants, WT plants cocultivated with *OxmiR399* plants in the same hydroponic tray and *OxmiR399* plants cocultivated with WT plants in the same hydroponic tray (*n* = 5 biological replicates). **c**, Expression of *SPL3 and SPL9* in WT plants, *OxmiR399* plants, WT plants cocultivated with *OxmiR399* plants in the same hydroponic tray and *OxmiR399* plants cocultivated with WT plants in the same hydroponic tray (*n* = 5 biological replicates). **d**, Luciferase activity in *pPHO2:Fluc* plants cocultivated with *OxmiR399* plants in the same hydroponic tray (*n* = 8 biological replicates). **e**, *Fluc* and *PHO2* mRNA levels in *pPHO2:Fluc* plants cocultivated with *OxmiR399* plants in the same hydroponic tray (*n* = 8 biological replicates). **f**, Luciferase activity in *pPHO2:Fluc* plants cocultivated with *35S:ami-Fluc* and *OxmiR399* plants in the same hydroponic tray (*n* = 8 biological replicates). **g**, *Fluc* and *PHO2* mRNA levels in *pPHO2:Fluc* plants cocultivated with *35S:ami-Fluc* and *OxmiR399* plants in the same hydroponic tray (*n* = 8 biological replicates). In the boxplots, dots represent single data points, whiskers denote minimum/maximum values, the box defines the interquartile range, the centre represents the median and box bounds represent the lower and upper quartiles. Welch’s *t*-test (two-sided) values are shown. Different letters (a,b,c) indicate differences in ANOVA tests (Tukey’s post‐hoc test, *P* < 0.05). Source data

Experimental evidence showing that exogenous miRNAs can influence gene expression in a receiving plant suggests the existence of exogenous miRNA in the environment. We grew wild-type and *35S:miR399* plants (*OxmiR399*) using a hydroponic system to check for the presence of miRNAs in the hydroponic solution. The results showed that *miR399d* is detectable at a higher level in the hydroponic solution in which *OxmiR399* plants were grown, and that wild-type plants secreted *miR399* (Fig. 7a).

To test whether the higher level of *miR399* secreted by *OxmiR399* plants could influence the expression of *PHO2* in wild-type plants grown nearby, we cocultivated *35S:miR399* and wild-type plants using the same hydroponic solution. Expression of *PHO2* was considerably lower in *OxmiR399* plants, but interestingly, also in wild-type plants cocultivated with *OxmiR399* plants (Fig. 7b). As a control, we analysed the expression of *SPL* genes, which are targets of *miR156*. Both *SPL3* and *SPL9* were expressed at a slightly lower level in *OxmiR399* plants compared with wild-type, but no influence of *OxmiR399* plants on the expression of *SPL* genes in wild-type plants was observed when both genotypes were grown in the same hydroponic tray (Fig. 7c).

We then cocultivated *pPHO2:Fluc* plants with *OxmiR399* plants. The results showed decreased LUC activity in *pPHO2:Fluc* plants that were cocultivated with *OxmiR399* plants (Fig. 7d). These results were corroborated by qPCR measurement of the mRNAs of *Fluc* and *PHO2*, both showing decreased expression levels in *pPHO2:Fluc* when cocultivated with *OxmiR399* plants (Fig. 7e). The experiment was repeated including a tray in which the *pPHO2:Fluc* plants were also cocultivated with plants that produced an artificial miRNA targeting the *Fluc* mRNA instead of the *miR399* target sequence in the *PHO2-UTR*. The results showed that cocultivation with either the *35S:ami-Fluc* (*OxamiFluc*, producing an artificial miRNA targeting the LUC sequence) or *OxmiR399* plants resulted in a substantial significant reduction in Fluc activity (Fig. 7f) as well as repression of *Fluc* and *PHO2* transcripts (Fig. 7g). Repression of the *Fluc* transcript was expected because both *miR399* and *ami-Fluc* target the *PHO2-UTR–Fluc* sequence in *pPHO2:Fluc* plants. Repression of *PHO2* after cocultivation with *OxmiR399* plants was similarly expected, whereas the slight repression of *PHO2* after cocultivation with *35S:ami-Fluc* plants suggests that secondary siRNAs are produced following cleavage of the *PHO2-UTR–Fluc* transcript by *ami-Fluc*. This indicated that the siRNA was able to recognize the *miR399* target sequence in the native *PHO2* transcript.

**Fig. 8:**
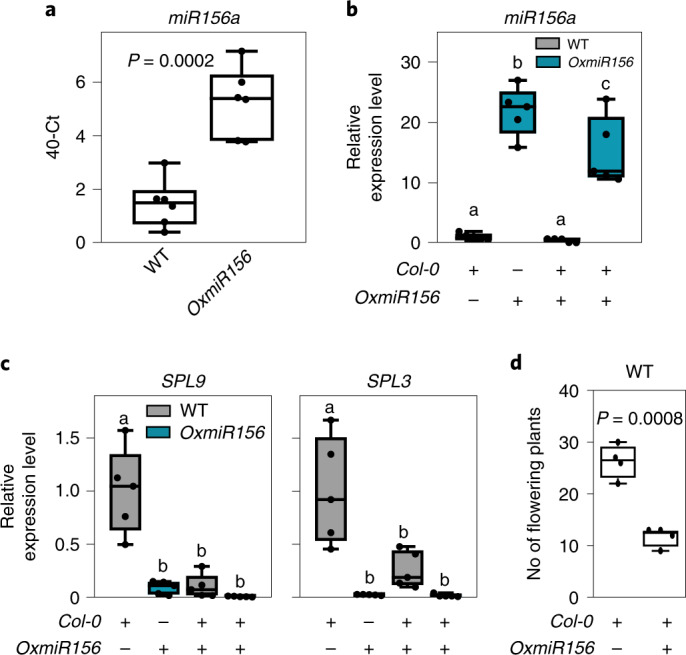
Plants overexpressing *miR156* influence the expression of *SPL* genes in WT plants sharing the same hydroponic medium. **a**, Presence and quantity of *miR156* in the external medium of WT and *OxmiR156* plants. **b**, *miR156* expression level in WT plants, *OxmiR156* plants, WT plants cocultivated with *OxmiR156* plants in the same hydroponic tray and *OxmiR156* plants cocultivated with WT plants in the same hydroponic tray. **c**, *SPL9* and *SPL3* expression level in WT plants, *OxmiR156* plants, WT plants cocultivated with *OxmiR156* plants in the same hydroponic tray and *OxmiR156* plants cocultivated with WT plants in the same hydroponic tray. **d**, Flowering of WT plants is delayed when cocultivated with *OxmiR156* plants. In the boxplots, dots represent single data points, whiskers denote minimum/maximum values, the box defines the interquartile range, the centre represents the median and box bounds represent the lower and upper quartiles. Welch’s *t*-test (two-sided) values are shown. Different letters (a,b,c) refer to differences in ANOVA tests (Tukey’s post‐hoc test, *P* < 0.05). **a**, *n* = 6 biological replicates; **b**,**c**, *n* = 5 biological replicates; **d**, *n* = 4 biological replicates, each represented by the mean value from a tray with 30 individual plants. Source data

*miR156a* was detected in the hydroponic medium in which plants overexpressing this miRNA were grown (Fig. 8a). A cocultivation experiment was performed, using wild-type and *35S:miR156* plants grown alone or mixed in the same hydroponic tray. The expression level of *miR156* itself was not influenced by cocultivation in either wild-type or *35S:miR156* plants (Fig. 8b). The expression of *SPL3* and *SPL9*, which are both targets of miR156, was considerably lower in wild-type plants that were grown together with *35S:miR156* plants (Fig. 8c). Interestingly, flowering of wild-type plants was strongly delayed when cocultivated with *35S:miR156* plants (Fig. 8d), which themselves flower extremely late due to a prolonged juvenile phase. These results indicate that miRNAs are secreted in the medium and that nearby plants are able to take up exogenous miRNAs. Expression of its target gene is therefore inhibited.

## Discussion

Plants can communicate with not only internal signalling mechanisms that include hormones, metabolic signals and sRNAs, but also other nearby organisms by emitting volatile organic compounds, volatile hormones such as ethylene and jasmonates. Plants also share a common mycorrhizal network with these organisms. To date, the possibility that sRNAs such as miRNAs represent not only mobile signalling molecules in the plant, but also signals among distinct plants has not been investigated. Studies have shown that exogenous RNAs can affect the plant response to pathogens (insects, viruses, fungi)^27–30^. In these experiments, dsRNAs of different lengths, designed and synthesized to target specific genes, were shown to be effective via an RNAi mechanism. The outcomes of infection by fungi and viruses as well as insect attacks can be reduced by exogenous RNA treatments as simple as leaf RNA sprays, highlighting enormous potential in agriculture^31^. Communication between plants and fungi has also been described, in which sRNAs synthesized by plants and pathogenic fungi are exchanged bidirectionally^30^.

Our results show that miRNAs are signalling molecules that enable communication between plants. This is supported by the following evidence. First, miRNAs are found in the external growth medium of *Arabidopsis* plants (Figs. 7 and 8). Second, in plants with lower expression levels of a specific miRNA, expression of the miRNA’s target gene(s) is affected by nearby plants overexpressing that specific miRNA (Figs. 7 and 8). We have shown that exogenous RNA extracts enriched in a specific miRNA can influence the expression of target gene(s) in a receiving plant or protoplast (Figs. 1, 3 and 4). Third, we have demonstrated that both miRNA-enriched RNA extracts and individual chemically synthesized miRNAs influence their targets when fed exogenously (Figs. 4–6).

Exactly how plants take up exogenous miRNAs is still unknown. The nematode *Caenorhabditis elegans* takes up external dsRNAs^16^, a mechanism requiring the SYSTEMIC RNA INTERFERENCE DEFECTIVE PROTEIN 2 (SID-2) transmembrane protein^32^. Uptake of exogenous dsRNAs in *C. elegans* is followed by activation of an RNAi response, specifically affecting gene expression. In mammals, exosomes contain a wide range of RNAs including miRNAs^33^, and are likely involved in the cell-to-cell transport of miRNAs, a mechanism that is operative in plants and in cross-kingdom RNAi^12^.

*Arabidopsis* cells secrete exosome-like extracellular vesicles to deliver sRNAs to the fungal pathogen, *B. cinerea*^12^. These sRNA-containing vesicles are taken up by the fungal cells and induce silencing of fungal genes which is critical for pathogenicity^12^. We ruled out the possibility that exogenous RNA fed to *Arabidopsis* plants, seedlings or protoplast enters as part of exosomes. This is because exosomes are likely to be destroyed by RNA extraction procedures, and naked, mature synthetic miRNA duplexes were able to influence expression of the target gene. It thus appears that uptake of naked miRNA molecules is possible in plants cells. Whether this is facilitated by a carrier protein, as in *C. elegans*, is still not clear.

Once inside the plant, exogenous miRNAs can generate a non-cell autonomous silencing signal^34^. In fact, miRNAs represent mobile signalling molecules by travelling systemically in plants^35^. The movement of miRNAs in plants is either short range, mostly through plasmodesmata, or long range, predominantly via the phloematic route^4,36^. In our experiments, exogenously fed Cy3-labelled *miR399* was found in the xylem (Fig. 3c,d). The xylematic route thus appears to be typical of exogenously fed RNAs, because exogenous RNA taken up through the petiole in *Nicotiana* was found to be strictly restricted to the xylem^37^. Endogenously produced miRNAs appear to follow a different route, given that an analysis of xylem and phloem exudate in *Brassica napus* revealed the presence of sRNAs in phloem sap, but not in the xylem^38^.

Systemic exogenous miRNA signalling is supported by evidence of repression of the *miR156* and *miR399* target genes in the leaves of plants that were cocultivated in the same hydroponic medium, thus indicating uptake from the root system and translocation, presumably via the xylem, to the aerial parts of the plant (Figs. 7 and 8). When exogenous RNAs were fed to intact young seedlings grown in a liquid medium, there was a much stronger response in the roots than the shoots (Fig. 1d). This suggests that the root system facilitates easier entry of miRNAs into the plant compared with the shoots. Interestingly, a miR156-dependent root phenotype^20,21^ is observed in plants fed with synthetic *miR156* (Fig. 2), indicating that modulation of *SPL* genes by exogenous *miR156* is strong enough to affect the phenotype of the receiving plant. Although *SPL10* was not repressed by the exogenous *miR156* treatment, both *SPL9* and, strongly, *SPL3* were repressed in the roots at the end of the experiment (Fig. 2f). Although *SPL10*, *SPL9* and *SPL3* were all assigned a function in defining the root architecture, single mutants for these *SPL* genes show enhanced secondary root production, indicating that they act non-redundantly^20^. The sole repression of *SPL3* by the exogenous *miR156* treatment is therefore sufficient to account for the observed phenotype. miRNA-targeted *SPL* genes are differentially expressed in *Arabidopsis* roots and this may account for the differential regulation by exogenous *miR156* (ref. ^20^).

The spread of silencing due to the translocation of exogenous miRNAs is amplified by the production of secondary siRNAs that rely on the activity of RDR6. In fact, silencing was found to be largely abolished in the *rdr6-15* mutant. AGO1 is required as well, indicating that exogenous miRNAs enter the canonical pathways of PTGS, with RDR6-dependent transitivity (Fig. 5).

Overall, our results showed that exogenous miRNAs exist outside the plant and have the potential to influence nearby plants by inducing PTGS in the receiving plant. Although this mechanism is probably distinct from the cross-kingdom exchange of sRNAs described between plants and pathogenic fungi, it highlights the existence of a common mechanism in which miRNAs represent signalling molecules that enable communication between plants. This may allow plants communities to respond to environmental conditions in a synchronized manner.

## Methods

### Plant material

*Arabidopsis thaliana* Columbia (*Col-0*) was used as the wild-type ecotype for all experiments unless differently stated. The *Arabidopsis* genotypes included the hypomorphic ARGONAUTE1 mutant *ago1-27* (ref. ^39^), the *rdr6-15* mutant^40^, the *rdr2-1* mutant^41^, *35S:mir399d*^6^ and *35S:mir156a*^8^. The *dcl1-9* and *hyl1* mutants were obtained from the Nottingham Arabidopsis Stock Centre (NASC; N3828 and N564863). *pSUC2:YFP* and *pS18:YFP* seeds were obtained from NASC (N2106198 and N2106196, respectively).

The two miRNA overexpressing lines are referred to as *OxmiR399* and *OxmiR156* in the text and figures. *Arabidopsis* seeds were vernalized at 4 °C in the dark for 48 h. They were then germinated at 22 °C day/18 °C night, with a photoperiod of 12 h. For soil-grown plants, seeds were sown directly on a 3:1 peat/perlite mixture. Plants growing in sterile conditions were obtained as follows: seeds were surface-sterilized using sequential incubations in 70% (v/v) ethanol, 5% (v/v) hypochlorite solution and then washed repeatedly with sterile water. For seedling experiments, seeds were sown in six multiwell plates containing 2 ml of liquid MS medium (Murashige and Skoog half-strength medium, 0.5% w/v sucrose, pH 5.7) under continuous shaking. For plants grown hydroponically, seeds were sown in polystyrene trays containing 30 rock-wool wells (Grodan) floating in hydroponic solution^42^.

### Treatment of seedlings with exogenous RNA

*Arabidopsis* wild-type and mutant seedlings were prepared as described above. Six-day-old seedlings were used for RNA feeding experiments. Before applying exogenous RNA, the media was removed and replaced by 2 ml of freshly prepared MS media. Total RNA, synthetic *ds-miR399*, synthetic *ds-miR319* or synthetic *ds-miR156* was added to the media as indicated in the figure legends. Seedlings (50 seedlings per well) were incubated with exogenous RNA completely submerged by the solution (with gentle shaking) as indicated in the figure legends.

### Confocal microscopy

Synthetic miRNA was labelled with Cy3 using the Silencer siRNA labelling kit (Thermo Fisher Scientific) in accordance with the manufacturer’s instructions. Five-day-old *Col-0* seedlings grown in vertical plates (MS half strength, 0.5% sucrose and 0.7% plant agar) were treated by applying 9.6 µl of the labelled miRNA (20 µM) along the roots. After 2 h incubation, confocal microscopy was performed. Samples were washed two or three times with distilled water to prevent labelled miRNA from attaching to the outer root surface. To verify that the fluorescence was emitted by the miRNA and not the free dye, a blank labelling reaction was performed (using water instead of miRNA), and plants were treated with this blank reaction. Treated roots were imaged using a Zeiss Airyscan 800 laser scanning confocal microscope. Cy3 miRNA was excited using a 561 nm laser and fluorescence was detected at 564–620 nm. *pSUC2:YFP* and *pS18:YFP* fluorescence was detected using a 488 nm laser and 492–550 nm emission wavelength. Sequential scanning was used to eliminate bleed-through.

### Cocultivation experiments

*Arabidopsis* plants were grown in polystyrene trays containing 30 rock-wool wells (Grodan) floating in hydroponic solution^42^. Plastic boxes (56 × 29 × 38 cm) containing 1 l of hydroponic solution were used. Wild-type and *35S:miR399d* plants were grown in separate trays/plastic boxes and also mixed in a shared tray/plastic box (cocultivation). Gene expression analysis was performed by harvesting 35-day-old plants. Five rosettes (five biological replicates) were used per treatment. In the experiment using *35S:mi156a* and wild-type plants, *35S:miR156a* plants were grown as described above for *35S:miR399d* plants.

### Constructs and transgenic line preparation

**Extended Data Fig. 8:**
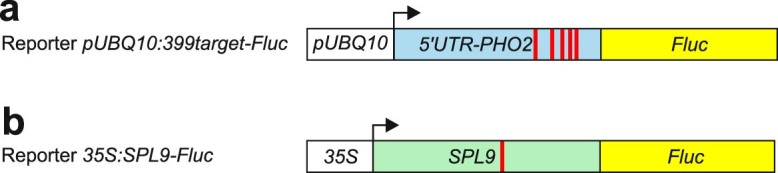
Schematic representation of the reporters. **a** *pUBQ10:PHO2-UTR-Fluc* and **b** reporter *35S:SPL9-Fluc*. Blue represents the 5’UTR, green the coding sequence, white the promoter, yellow the *Fluc* coding sequence. The red bars identify the miRNA target sites (five sites in the 5’UTR of *PHO2*, one site in the *SPL9* cds).

*Arabidopsis* *miR399* triggers repression of *PHO2* mRNA targeting five complementary sequences located in the 5′-UTR of the *PHO2* transcript. We produced a plasmid named *pUBQ10:PHO2-UTR–Fluc*, which expresses a *Fluc* mRNA fused to 5′-UTR of *PHO2* (Extended Data Fig. 8). The 5′-UTR of *PHO2* was fused to the promoter of *UBIQUITIN10* by overlapping PCR using Phusion High-Fidelity DNA Polymerase (Thermo Fisher Scientific), and then introduced into the pENTR/D‐topo vector (Thermo Fisher Scientific) to generate *pENTR‐pUBQ10:5*′*UTR*_*PHO2*_. The resulting entry vector was recombined into the destination vector pGWL7 using the LR reaction mix II (Thermo Fisher Scientific) to obtain the expression vector *pUBQ10:PHO2-UTR–Fluc*. A mutated version of plasmid *pUBQ10:PHO2-UTR–Fluc* was created by replacing part of the wild-type sequence of the 5′-UTR of *PHO2* with a sequence lacking the *miR399* target sequences (Extended Data Fig. 3). The mutated oligonucleotide was generated synthetically (GeneArt, Thermo Fisher Scientific) and inserted by ligation into *pENTR‐pUBQ10:5*′*UTR*_*PHO2*_. The entry vector was recombined into the destination vector pGWL7, as described above. *Ath-miR156a* targets the coding sequence of *SPL9*. The *SPL9* coding sequence was cloned from cDNA using Phusion High-Fidelity DNA Polymerase (Thermo Fisher Scientific), subcloned in pENTR/D‐topo vector and then recombined into p2GWL7 by LR clonase (Thermo Fisher Scientific) to obtain the expression vector *35S:SPL9*, which was used to produce the *35S:SPL9-Fluc* transgenic line (Extended Data Fig. 8).

**Extended Data Fig. 9:**
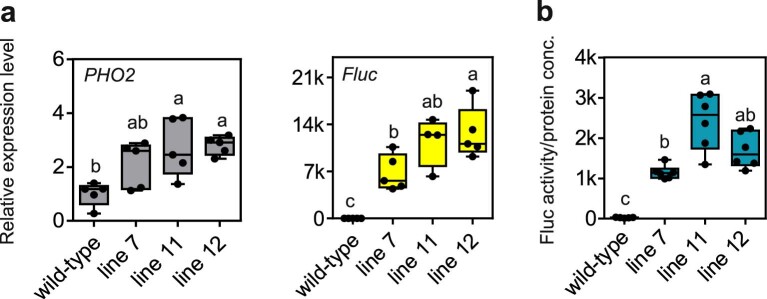
Screening of transgenic *pPHO2:399target-Fluc* seedlings. Screening of transgenic *pPHO2:399target-Fluc* seedlings for RNA expression level **a** and the activity of luciferase **b**. a: Data are mean of 6 biological replicates ± SD. b: The bottom and top of each box denote the first and third quartile, respectively (n = 6). In the box-blots, the dots represent the single data points, whiskers denote the min/max values, the box defines the interquartile range, centre represents the median, box bounds represent the lower and upper quartiles. Different letters (a,b,c) refer to differences in ANOVA tests (Tukey’s post‐hoc test, P < 0.05). Source data

To produce transgenic plants bearing the *pPHO2:Fluc* construct, a 3.5-kb sequence upstream of the *PHO2* start codon was PCR amplified from genomic DNA using Phusion High-Fidelity DNA Polymerase (Thermo Fisher Scientific) and cloned in the pENTR/D‐topo vector (Thermo Fisher Scientific). Subsequently, the *PHO2* promoter was recombined into pBGWL7 by LR clonase (Thermo Fisher Scientific). The final vector was transformed into *Arabidopsis* *Col-0* plants via *Agrobacterium tumefaciens*-mediated transformation, using the floral dip method. Transgenic seedlings were screened for glufosinate ammonium resistance. Independent lines were selected measuring the RNA expression level and the activity of luciferase as described above (Extended Data Fig. 9). The *pPHO2:PHO2-UTR–Fluc* reporter line was crossed with *ago1-27* and *rdr6-15* mutants.

The *ami-Fluc* construct used in this study was designed as follows. The 21-nucleotide sequence of the mature miR319a was replaced in the pre-miR319a sequence by the 21-nucleotide sequence targeting the firefly luciferase transcript (TTAACGCCCAGCGTTTTCCCG). Artificial microRNA targeting the Firefly reporter gene was designed as described in Schwab et al.^43^. The region in the miRNA base pairing with the *ami-Fluc* 21-nucleotide sequence along the hairpin structure of the pre-miRNA with the sequence was edited to maintain perfect pairing (Supplementary Table 1)^43^. A gateway entry vector containing the above described *ami-Fluc* flanked by *attL1* and *attL2* sites was synthesized using GeneArt (Thermo Fisher Scientific). The entry vector was subsequently used in the LR reaction with the pB7WG2 destination vector^44^ to generate the final *35S:ami-Fluc* construct. *Agrobacterium* GV3101 strain was used to deliver the T-DNA into the *Arabidopsis* genome via floral dip. Transgenic T1 seeds were stratified on MS1/2 agar media containing 25 µg ml^−1^ of Basta (glufosinate ammonium) for selection of transgenic seedlings. A full list of primers used in the cloning is reported in Supplementary Table 2. *ami-Fluc* expression was assessed by qPCR using the primers reported in Supplementary Table 3.

### Protoplast isolation and transformation

*Arabidopsis* mesophyll protoplasts were isolated and transformed as described by Iacopino et al.^45^. Protoplasts were isolated from leaves of 3-week-old plants through incubation in enzymatic solution (1% w/v cellulase, 0.3% w/v macerozyme, 0.4 M mannitol, 20 mM KCl, 10 mM CaCl_2_, 20 mM MES, pH 5.7) for 3 h in the dark at 22 °C. Afterwards, protoplasts were filtered, washed twice with W5 solution (154 mM NaCl, 125 mM CaCl_2_, 5 mM KCl, 2 mM MES, pH 5.7) and subsequently centrifuged for 2 min at 100 × *g* before being resuspended in 0.4 M mannitol, 15 mM MgCl_2_, 4 mM MES (pH 5.7) until a final concentration of 5 × 10^5^ cells ml^−1^ was obtained. For the transformation, 4 µg of each plasmid was added to 100 µl of protoplast suspension and then mixed gently with an equal volume of a 40% PEG 4000 solution (0.2 M mannitol, 100 mM CaCl_2_). The mixture was incubated for 20 min at room temperature in the dark, and then 440 µl of W5 solution was added to stop the transformation. Protoplasts were centrifuged at 100 × *g*. for 2 min, resuspended in 1 ml of WI solution (50 mM mannitol, 4 mM MES pH 5.7, 20 mM KCl, 50 mM glucose) and transferred to six multiwell plates. For the exogenous RNA feeding experiments, 1 µl of total RNA (0.1 µg µl^−1^) isolated from *35S:miR399d* plants or 1 µl of synthetic ds-miRNA (0.2 µM) was added to the WI solution. Protoplasts were incubated overnight at 22 °C in the dark. For luciferase activity quantification, protoplasts were pelleted by centrifugation at 4,000 × *g* for 1 min, and flash frozen in liquid nitrogen.

### Luciferase activity quantification

Firefly (*Photinus pyralis*) and *Renilla reniformis* luciferase activities were quantified using the Dual-Luciferase Reporter Assay System (Promega) following the manufacturer’s instructions. For protoplast transient transformation, activity of firefly luciferase was normalized on *Renilla* luciferase activity. For transgenic plants, total proteins were extracted with Passive Lysis buffer (Promega). Firefly luciferase activity was normalized on protein concentration which was quantified using Bradford protein assay (Bio-Rad).

### Total RNA extraction and real-time qPCR analysis

Total RNA was extracted from *Arabidopsis* seedlings using a phenol–chloroform extraction protocol^46^. RNA quality was assessed by electrophoresis using a 1% (w/v) agarose gel, followed by spectrophotometric quantification. cDNA was synthesized from 1 µg of total RNA using the Maxima First Strand cDNA Synthesis Kit for RT–qPCR, with dsDNase (Thermo Fisher Scientific). For real-time qPCR, 30 ng of cDNA were analysed using an ABI Prism 7300 sequence detection system (Applied Biosystems). PowerUp SYBR Green Master Mix (Thermo Fisher Scientific) was used to monitor dsDNA synthesis in accordance with the manufacturer’s instructions. Expression of UBIQUITIN10 (*At4G05320*) was used as the housekeeping gene for internal normalization. Relative RNA levels were calculated using GeNorm (http://medgen.ugent.be/~jvdesomp/genorm).

Mature miRNAs were quantified using the stem–loop RT–PCR technique^47^. A total of 2.75 µg of total RNA was subjected to DNase treatment using DNA RQ1 RNase-free DNase (Promega), and cDNA was synthesized using Superscript IV (Invitrogen) according to the manufacturer’s instructions. qPCR amplification was performed on 50 ng of cDNA, as described above. Supplementary Tables 3 and 4 give a list of the primers used for qPCR analysis.

### Synthetic miRNAs, annealing of RNA oligonucleotides and labelling reaction

*Ath-miR399d* and *Ath-miR156a* sequences were obtained from the miRBase database (www.mirbase.org). Mature miRNA sequences and their complementary strands were chemically synthesized by Eurofins genomics (https://www.eurofinsgenomics.eu/). The nucleotide sequence (5′ to 3′) for the miRNAs used are reported in Supplementary Table 1. The 3′-end of the strands carried a methyl group, as did the endogenous miRNAs. The strands were resuspended in sigma water. A purification step was then performed using Illustra MicroSpin G-25 columns (GE Healthcare) to remove possible contaminants from the strands. The annealing reaction was performed by combining 50 µM solutions of mature miRNA and passenger strand with 5× annealing buffer (Thermo Fisher Scientific) for a final concentration of 20 µM. The mixture was incubated at 90 °C for 1 min, followed by a gradual decrease in temperature to 37 °C, held for 45 min. RNA oligonucleotides were labelled using the Silencer siRNA Labelling Kit with Cy 3 dye (Thermo Fisher Scientific) according to the manufacturer’s instructions. Labelling of dsRNA was confirmed by electrophoresis using 1% (w/v) agarose gel followed by visualization using a ChemiDoc XRS+ imaging system (Bio-Rad).

### 5′-RACE–PCR experiment

For the detection of 3′-cleavage products from *miR399*-targeted *Fluc*, 5′-RACE was performed using the FirstChoice RLM-RACE Kit (Invitrogen). Seedlings were grown in six multiwell plates containing 2 ml of liquid MS medium under continuous shaking. Six-day-old seedlings were used for RNA feeding experiments. Before applying exogenous RNA, the medium was removed and replaced by 2 ml of freshly prepared MS media. To detect the cleavage products of *miR399*-targeted *Fluc* transcript, synthetic *ds-miR399* was added to the media to a final concentration of 0.2 nM ml^−1^. After 72 h of incubation with the RNA, seedlings were collected in liquid nitrogen and total RNA was extracted with a Spectrum Plant Total RNA Kit (Sigma). A total of 1 μg of total RNA was ligated directly with the 5′-RACE Adapter oligonucleotide without further processing of the RNA samples. Reverse transcription was carried out according to the manufacturer’s instructions. Semiquantitative PCR reactions were performed to quantify 3′-cleavage products and full-length transcripts. The PCR reactions were performed under regular conditions: 35 cycles of 94 °C for 30 s, 60 °C for 30 s and 72 °C for 1 min. The same amount of RNA was reverse transcribed with random decamers to amplify UBQ10 as an internal loading control. Oligonucleotide sequences for PCR amplification are listed in Supplementary Table 5. The 3′-cleavage products of PHO2 were subcloned into a pCR 2.1 vector (Thermo Fisher Scientific) and sequenced to detect the cleavage site of miR399d on the 5′-UTR of PHO2.

### Statistical analyses

All data are from biological replicates. Data were analysed using one- and two-way analysis of variance (ANOVA) tests (Tukey’s post‐hoc test; different letters in the figures indicate differences for *P* < 0.05; individual *P* values are provided in the source data files), and different letters were assigned to values that differed significantly from each other. Pairwise comparison was performed by Welch’s *t*-test (actual *P* values are given in the figures). In the boxplots, dots represent single data points, whiskers denote minimum/maximum values, the box defines the interquartile range, the centre represents the median and box bounds represent the lower and upper quartiles. GraphPad v.8 was used to perform statistical analysis of the data.

### Reporting Summary

Further information on the research design is available in the Nature Research Reporting Summary linked to this article.

## Supplementary information

Supplementary Information Supplementary Tables 1–5.

Reporting Summary

## Data Availability

There are no restrictions on data availability. All data are reported in the figures, in the supplementary material file and in source data files. The miRNA database utilized is miRbase: https://www.mirbase.org/. Source data are provided with this paper.

## Source data

Source Data Fig. 1 Graph data and single *P* values.
Source Data Fig. 2 Graph data.
Source Data Fig. 3 Graph data and single *P* values.
Source Data Fig. 4 Graph data and single *P* values.
Source Data Fig. 5 Graph data.
Source Data Fig. 6 Graph data and single *P* values.
Source Data Fig. 7 Graph data and single *P* values.
Source Data Fig. 8 Graph data and single *P* values.
Source Data Extended Data Fig. 1 Graph data and single *P* values.
Source Data Extended Data Fig. 2 Graph data and single *P* values.
Source Data Extended Data Fig. 4 Unprocessed gel images.
Source Data Extended Data Fig. 5 Graph data and single *P* values.
Source Data Extended Data Fig. 6 Graph data and single *P* values.
Source Data Extended Data Fig. 7 Graph data.
Source Data Extended Data Fig. 9 Graph data and single *P* values.
