# Supplementary material for: Exogenous miRNAs induce post-transcriptional gene silencing in plants
Source: Nat Plants. 2021 Oct 14;7(10):1379–88. doi: 10.1038/s41477-021-01005-w (PMC8516643; doi:10.1038/s41477-021-01005-w)
Supplement: Supplementary file 1 — Supplementary Tables 1–5. [file 41477_2021_1005_MOESM1_ESM.pdf]

nature portfolio

https://doi.org/10.1038/s41477-021-01005-w

Supplementary information

Exogenous miRNAs induce post-transcriptional gene silencing in plants

In the format provided by the authors and unedited

Supplementary Information

Exogenous miRNAs induce post-transcriptional gene silencing in plants

Federico Betti[1*], Maria Jose Ladera-Carmona[1*], Daan A. Weits[1], Gianmarco Ferri[2], Sergio Iacopino[3], Giacomo Novi[1], Benedetta Svezia[1], Alicja Barbara Kunkowska[1], Antonietta Santaniello[4], Alberto Piaggesi[4], Elena Loreti[5**], Pierdomenico Perata[1**]

[1]PlantLab, Institute of Life Sciences, Sant'Anna School of Advanced Studies, Pisa, Italy; [2] NEST, Scuola Normale Superiore, Pisa, Italy; [3]Department of Biology, University of Pisa, Pisa, Italy, [4]Valagro spa, Atessa (CH), Italy; [5]Institute of Agricultural Biology and Biotechnology, National Research Council, Pisa, Italy

Table S1. *amiFluc* complete sequence and oligonucleotide sequences (5'→3') of artificial miRNAs.

amiFluc complete sequence (underlined: Fluc targeting sequence; bold: edited complementary strand in the hairpin)	
amiFluc	CAAACACACGCTCGGACGCATATTACACATGTTCATACACTTAATACTCGCTGTTTTGAATTGATGTTTTAGGAATATATATGTAGACGAGAAAACGCTGCGCGTTATTCACAGGTCGTGATATGATTCAATTAGCTTCCGACTCATTCATCCAAATACCGAGTCGCCAAAATTCAAACTAGACTCGTTAAATGAATGAATGATGCGGTAGACAAATTGGATCATTGATTCTCTTTGA**TTAACGCCCAGCGTTTTCCCG**TCTCTCTTTTGTATTCCAATTTTCTTGATTAATCTTTCCTGCACAAAAACATGCTTGATCCACTAAGTGACATATATGCTGCCTTCGTATATATAGTTCTGGTAAAATTAACATTTTGGGTTTATCTTTATTTAAGGCATCGCCATG

miRNA	Mature strand	Complementary strand
miR399d	UGCCAAAGGAGAUUUGCCCCG	UACUGGGCGAAUACUCCUAUGG
miR156a	UGACAGAAGAGAGUGAGCAC	GCUCACUGCUCUUUCUGUCAGA
miR319b	UUGGACUGAAGGGAGCUCCCU	AGAGCUUUCUUCGGUCCACUC

Table S2. List of oligonucleotides used for cloning.

Name	Sequence (5'→3')
pUBQ10-Fw	CACCGTCATTATCCTATGCAAATC
pUBQ10-5'UTR$_{PHO2}$-Fw	CACACACGAGGTGGGATACT
pUBQ10-5'UTR $_{PHO2}$-Rv	AGTATCCCACCTCGTGTGTG
5'UTR $_{PHO2}$-Rv	AGTTTAGACGCCTGCCATGGAACTG
SPL9-Fw	CACCATGGAGATGGGTTCCAAC
SPL9-Rv	GAGACCAGTTGGTATGGTGAGAAG
pPHO2-Fw	CACCAACCAGAACTAGATCGCGTCA
pPHO2-Rv	AGTTTAGACGCCTGCCATGGAACTG

Table S3. Stem-Loop primers used for miRNA expression analysis using RT-qPCR.

miRNA	Stem-Loop primer	Forward primer (5'→3')	Universal Reverse primer (5'→3')
miR156a	GTCGTATCCAGTGCAGGGTCCGAGGTATTC GCACTGGATACGACGTGCTC	GCGGCGGTGACAGAAGAGAGT	GTGCAGGGTCCGAGGT
miR399d	GTCGTATCCAGTGCAGGGTCCGAGGTATTC GCACTGGATACGACCGGGGCAAA	CGACGTGCCAAAGGAGATTTG	
*miR399**	GTTGGCTCTGGTGCAGGGTCCGAGGTA TTCGCAC CAGAGCCAACCCATAG	GTGTACTGGGCGAATACTC	
amiFluc	GTCGTATCCAGTGCAGGGTCCGAGGTATTC GCACTGGATACGACCGGGAA	GCGGCGGTTAACGCCCAGCGT	

Table S4. List of primer sequences used for qPCR analysis.

Name	Forward primer (5'→3')	Reverse primer (5'→3')
At4g05320 (UBQ10)	GGCCTTGTATAATCCCTGATGAATAAG	AAAGAGATAACAGGAACGGAAACATAGT
At2g33770 (PHO2)	AGCTGACCGTTGCTGTCAGTC	CGGTGTAGCTTCCCTGTTTGTT
At2g33810 (SPL3)	CTTAGCTGGACACAACGAGAGAAGGC	GAGAAACAGACAGAGACACAGAGGA
At2g42200 (SPL9)	TGTGGCTGGTATCGAACAGAGG	TTCCGGAAGCTGATGAAACCTG
At1G27370(SPL10)	GTGGGAGAATGCTCAGGAGGC	GAGTGTGTTTGATCCCTTGTGAATCC
5'UTR$_{PHO2}$Fluc	TTCCATGGCAGGCGTCTAAA	AGGAACCAGGGCGTATCTCT

Table S5. Oligonucleotide sequences used for 5' RACE-PCR.

Name	Forward primer (5'→3')
5' RACE Adapter	CUGAUGGCGAUGAAUGAACACUGCGUUUGCUGGCUUUGAUGAAA
5'RACE Outer primer	GCTGATGGCGATGAATGAACACTG
5'RACE Inner primer	CGCGGATCCGAACACTGCGTTTGCTGGCTTTGATG
UBQ10 F	GGCCTTGTATAATCCCTGATGAATAAG
UBQ10 F	AAAGAGATAACAGGAACGGAAACATAGT
5'UTR_{PHO2} F1	CTGTTTCCCATTTATACTTCAGATTC
FLUC R1	CGTTCATTATAAATGTCGTTCGC
FLUC R2	AGGAACCAGGGCGTATCTCT
