# Supplementary figures and images for: Exogenous miRNAs induce post-transcriptional gene silencing in plants
Source: Nat Plants. 2021 Oct 14;7(10):1379–88. doi: 10.1038/s41477-021-01005-w (PMC8516643; doi:10.1038/s41477-021-01005-w)

Source data for Extended data Figure 4a

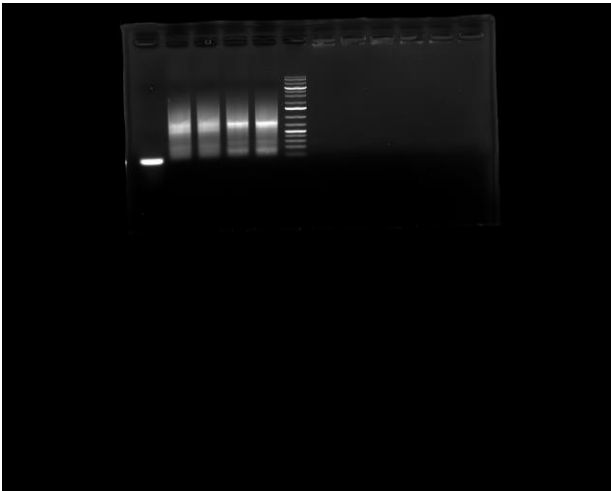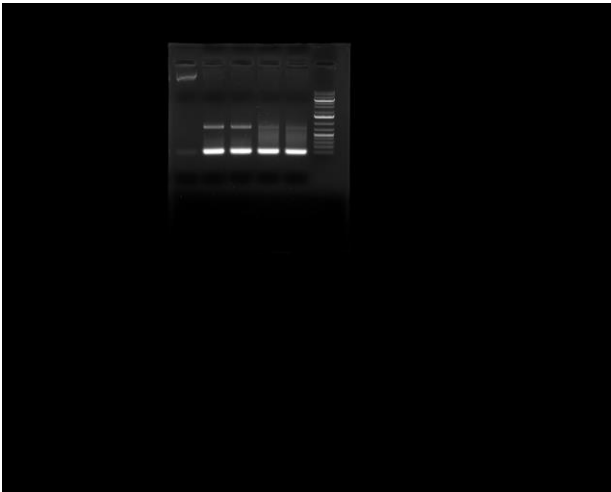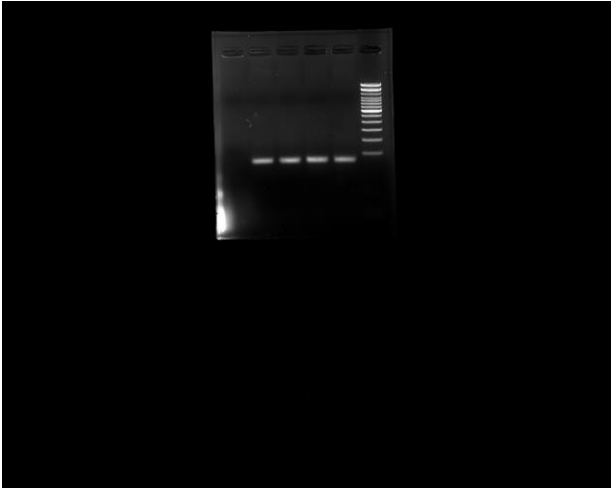

Supplement: Source Data Extended Data Fig. 4 — Unprocessed gel images. [file 41477_2021_1005_MOESM13_ESM.pdf]
